# Case Report: Dilated cardiomyopathy with biventricular thrombus secondary to impaired coagulation in a patient with HIV

**DOI:** 10.12688/f1000research.24016.2

**Published:** 2020-07-07

**Authors:** Chetan Brahma Kammari, Suhasini Rallabandi, Harsha Rallabandi, Subba Rao Daggubati, Sreedhar Adapa, Srikanth Naramala, Venu Madhav Konala

**Affiliations:** 1 Department of Internal Medicine, Cape Fear Valley Hospital, Fayetteville, NC, 28304, USA; 2 Department of Internal Medicine, Mery Hospital Joplin, Joplin, MO, 64804, USA; 3 Gandhi Medical College, Secunderabad, Telangana, 500003, India; 4 Wise Health System, Decatur, TX, 76234, USA; 5 Department of Internal Medicine, Division of Nephrology, Kaweah Delta Medical Center, Visalia, CA, 93291, USA; 6 Department of Internal Medicine, Division of Rheumatology, Adventist Medical Center, Hanford, CA, 93230, USA; 7 Department of Internal Medicine, Division of Medical Oncology, Ashland Bellefonte Cancer Center, Ashland, KY, 41101, USA

**Keywords:** HIV, Hypercoagulable, Ventricular, thrombus, protein c, protein s, antithrombin 3

## Abstract

Human immunodeficiency virus (HIV) infection is a known hypercoagulable state with venous thromboembolism with a high mortality rate compared to the general population. The homeostatic balance in HIV infected patients improves with treatment compared to those who are not.  A decreased hypercoagulable state noted by low levels of Von Willebrand factor, factor VIII and d-dimer levels along with higher protein C and S activity in patients on treatment suggests that hypercoagulable state is partially correctable with highly active antiretroviral therapy.  HIV with heart muscle involvement can present as myocarditis or as dilated cardiomyopathy with left or right ventricular dysfunction.  Here we present a case of a 57-year-old man with a known history of HIV infection, noncompliant with medical therapy presenting with dilated cardiomyopathy with biventricular thrombi with reduced protein C, protein S, and Antithrombin III levels.

## Introduction

Human immunodeficiency virus (HIV) infection is a well-known hypercoagulable state associated with venous thromboembolism with high mortality risk compared to the general population ^1, 2^. HIV with heart muscle involvement can present as myocarditis or as dilated cardiomyopathy with left or right ventricular dysfunction ^3^. Here we present a case of a patient infected with HIV presenting with dilated cardiomyopathy with biventricular thrombi secondary to reduced protein C, protein S, and antithrombin III levels. On review of the literature, we were able to find only one similar presentation where a patient with HIV has cardiomyopathy with biventricular thrombosis ^4^.

## Case report

The patient is a 57-year-old Caucasian male with a known past medical history of the human immunodeficiency virus (HIV) non-compliant with medical therapy and hyperlipidemia, who presented to the emergency department with shortness of breath, hypoxia with oxygen saturation of 70%, pleuritic chest pain and a syncopal episode with fall. The patient denied any significant family, surgical, or social history. He was treated for pneumonia six weeks before presentation with antibiotics, and since then, he has been experiencing exertional dyspnea. Patient unable to do his activities of daily living due to exertional dyspnea. The patient denied orthopnea or paroxysmal nocturnal dyspnea. He had a syncopal episode at home with fall resulting in left pleuritic chest pain. The patient admitted that he had previous syncope episodes that occur with little or no warning signs except for mild dizziness before passing out. The physical examination was significant for chest wall tenderness with a normal cardiorespiratory exam.

**Figure 1.**
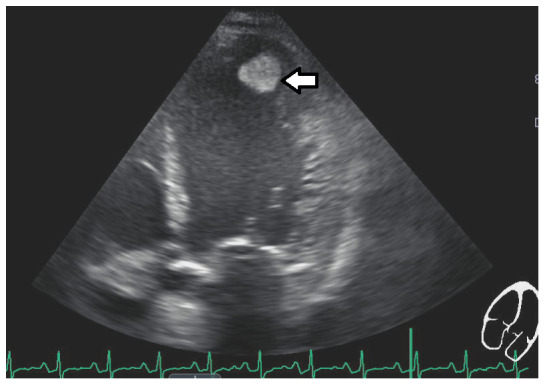
Echocardiogram (Apical 2 chamber view) showing dilated left ventricle showing apical thrombus.

Laboratory findings showed mildly elevated troponin. An echocardiogram demonstrated biventricular dilatation with ejection fraction (EF) of 30% and compelling evidence for the presence of thrombus in the apex of both ventricles and free wall of the right ventricle (as shown in Figure 1– Figure 4). Echocardiogram did not demonstrate any spontaneous echo contrast, suggesting severely diminished ejection fraction and stagnation of blood flow. Orthostatic vitals were normal, and the patient did not experience any arrhythmias on telemetry ruling them out as a cause for syncope. Syncope was later presumed to be likely secondary to a low flow state from reduced EF. The patient denied any prior history of deep vein thrombosis, transient ischemic attack, or stroke. CT chest with contrast did not show any evidence of pulmonary embolism but showed diffuse cardiomegaly ( Figure 5 and Figure 6). Given the presence of biventricular thrombus, the patient was evaluated for the hypercoagulable state. Results showed low Protein C, protein S, and antithrombin III levels. Factor V Leiden and lupus anticoagulant were normal. The laboratory findings are summarized in Table 1.

**Figure 2.**
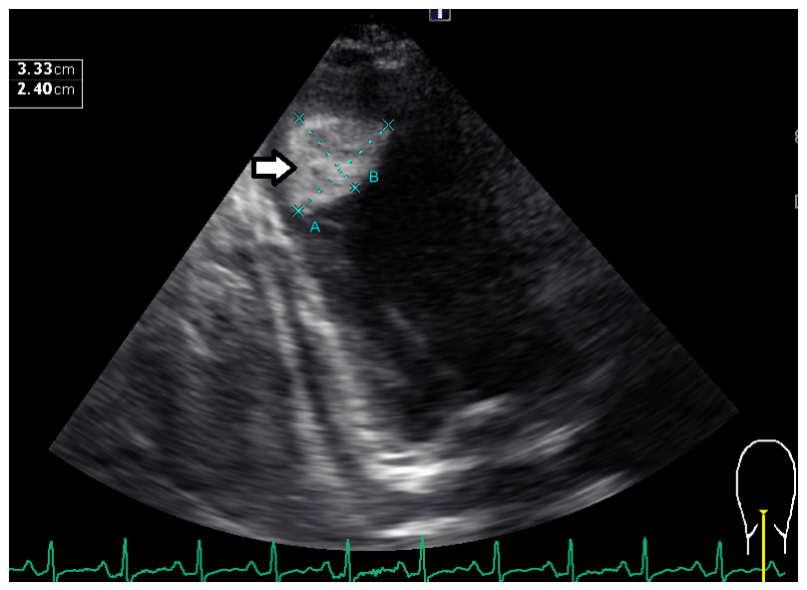
Echocardiogram (Apical 2 chamber view) showing dilated left ventricle with apical thrombus measurements.

**Figure 3.**
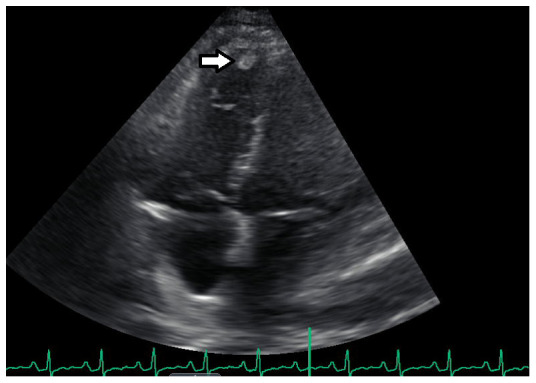
Echocardiogram (Apical 4 chamber view) showing dilated right ventricle with apical thrombus.

**Figure 4.**
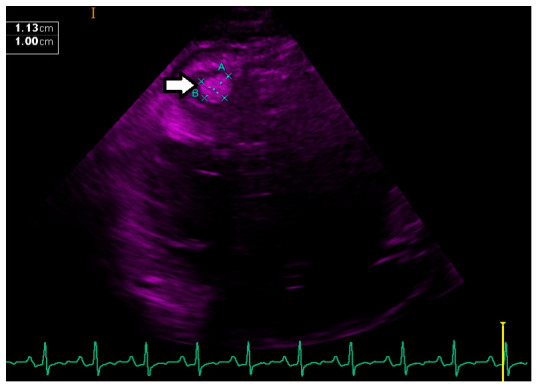
Echocardiogram (Apical 4 chamber view) showing dilated right ventricle with apical thrombus measurements.

**Figure 5.**
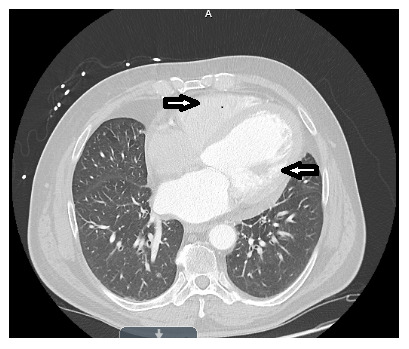
CT of chest with IV contrast showing left ventricular and right ventricular enlargement.

**Figure 6.**
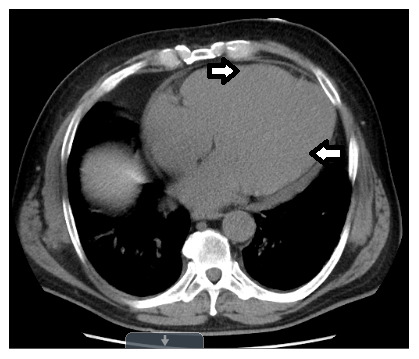
CT of chest without contrast showing left ventricular and right ventricular enlargement.

**Table 1.** Summary of laboratory findings.

	Patient value (normal range)
Sodium	132 mol/L (136–145)
Glucose	64 mg/dl (74–99)
Blood Urea Nitrogen	31 mg/dl (6–20)
Creatinine	1.09 mg/dl (0.7–1.20)
White Blood Cell Count	13.4 k/ul (4.5–0.8)
Absolute Neutrophil Count	8.65 k/ul (1.5–7)
Hemoglobin	16.5 g/dl (13.5–18.0)
Platelets	346 k/ul (150–450)
HIV Antibodies	Positive
CD4 T Cell Count	815cells/mcl (365–1437)
CD8 T Cell Count	614 cells/mcl (117–846)
CD4/CD8 Ratio	1.3 (>0.9)
Hepatitis A Antibody	Negative
Hepatitis B Surface and Core Antigen	Negative
Hepatitis C Antibody	Negative
Alkaline Phosphatase	152 u/l (40–129)
Aspartate Aminotransferase	270 u/l (10–50)
Alanine Aminotransferase	458 u/l (10–50)
NT pro B-type Natriuretic Peptide	4485 pg/ml (0–125)
Troponin (0 hr, 6 hr, 12 hr)	89, >90, >106 ng/l (<15)
Protein C Activity	46% (70–140)
Protein S Activity	38% (45–125)
Antithrombin III Activity	77% (80–120)
Factor V Leiden	Negative
Lupus Anticoagulant	Negative
Anti-Cardiolipin Antibodies	Normal
Anti-Beta 2 Glycoprotein Antibodies	Normal
Prothrombin Gene Mutation	Negative

Cardiology initially considered cardiac catheterization to delineate the patient’s coronary anatomy for the potential need for revascularization, but instead decided to perform stress myocardial perfusion study to prevent thromboembolic events and strokes that can be associated with the procedure. The nuclear stress test was negative for reversible ischemia. There was no evidence of fixed defects on the stress test, suggesting no evidence of prior myocardial infarction or scar tissue within the myocardium. The patient was started on Lasix 20mg oral daily, metoprolol succinate 25mg oral daily, and low-dose Lisinopril 2.5mg oral daily. Novel anticoagulants (NOACs) are not recommended for mural thrombus; therefore, the patient was started on a therapeutic dose of low molecular weight heparin 60mcg subcutaneous twice daily and bridged with warfarin 5mg oral daily. Heparin was discontinued once his International Normalized Ratio (INR) was therapeutic. One week after the admission, the patient was ready to be discharged. His symptoms had significantly improved with no further syncopal episodes or exertional dyspnea. His ambulatory oxygen saturations were normal on room air. The patient was advised to follow up with infectious diseases to initiate highly active antiretroviral therapy (HAART) therapy for his HIV, cardiology and hematology for continued care.

## Discussion

HIV infection is a well-known hypercoagulable state with a frequency of thrombotic events recognized in the range of 0.19% to 7.63% per year ^1^. Compared to the general population of the same age, the risk of arterial and venous thrombosis in HIV infected patients is increased two to tenfold. One possible explanation could be due to the presence of multiple comorbidities and baseline increased inflammatory/hypercoagulable state. Risk factors like low CD4 cell count, especially in the presence of clinical acute immunodeficiency syndrome (AIDS), protein S deficiency, and protein C deficiency, have demonstrated the strongest association with venous thromboembolism. Other less significant and controversial risk factors include protease inhibitor therapy, opportunistic infections, and positive antiphospholipid antibodies, including anticardiolipin antibodies and lupus anticoagulant ^1, 5^. Thrombophilic abnormalities in total platelet count, protein C, and S activity are directly correlated to CD4 count ^6^, and their frequency increases as the patient progresses to AIDS ^7^. HIV infection with venous thromboembolism has a high mortality rate compared to the general population ^2^.

The homeostatic balance in HIV-infected patients varies if they are on HAART therapy vs. not being on treatment. The indicators of hypercoagulable state like the activity of the Von Willebrand factor, levels of Factor VIII, and D dimer are lower in patients on treatment. Also, anticoagulant proteins like protein C and S activity are higher in patients on therapy, suggesting the hypercoagulable state is partially correctable with HAART. Although patients on HAART therapy have a lower prevalence of coagulation abnormalities, many show the persistent procoagulant state as evident by increased endothelial cell activation and high APCsr when compared to the general population. Continued coagulation markers abnormalities have been observed in HIV-infected individuals before and after the initiation of HAART ^8^. Even patients who are newly started on HAART therapy showed marked improvement in the coagulation profile but still different from the general population, indicating a persistent abnormal homeostatic balance ^9^. In addition, it has been previously observed that most HIV positive patients with low protein S levels also had mutations in exon 15 of PROS 1 gene warranting further investigation ^10^.

Cardiac dysfunction in HIV-infected patients evidenced by congestive heart failure is a well-documented finding. Even in asymptomatic HIV patients, an echocardiographic and echo-doppler examination has shown evidence of early signs of impaired systolic and diastolic function, suggesting an early involvement of the heart in HIV disease ^11^. Cardiomyopathy associated with HIV may be related to the autoimmune process induced by HIV in conjugation with other cardio tropic virus or could be direct action on the heart muscle ^12^. It is shown that the incidence of dilated cardiomyopathy in HIV is 17.6% ^13^. HIV with heart muscle involvement can present as myocarditis or dilated cardiomyopathy with left or right ventricular dysfunction, the pathogenesis of which seems to relate to the coinfection with other infectious agents ^3^. Isolated right and left heart dysfunction have no direct correlation to the CD4 count and does not carry adverse prognostic implications ^14^.

Long term anticoagulation may be beneficial in HIV infected patients to prevent future thromboembolic events as most of the contributing risk factors are often irreversible. Since there is a possibility of interactions between warfarin and antiretroviral therapy, health care providers should be watchful of consequent dangerously high or low INRs when giving warfarin to patients undergoing antiretroviral therapy ^1^. It is shown that newer anticoagulants can be used with antiretroviral therapy without any noticeable interactions ^15^.

## Conclusion

In our patient, a bi-ventricular thrombus is likely the result of the hypercoagulable state along with severe ventricular dysfunction with dilated cardiomyopathy due to underlying HIV infection. Systemic or pulmonary embolization was not seen in our patient, as reported in the past as an associated finding ^16^. Physicians caring for patients with HIV should always consider thrombotic and thromboembolic events in the differential besides known malignancies and opportunistic infections when treating patients with unexplained dyspnea or hypoxia, especially in young males.

We think that low protein C, low protein S levels, and antithrombin-III deficiency could contribute to thrombus formation if truly positive in addition to the risk factors discussed above. However, it could represent a thrombus consuming the factors, and they will be repeated six months to confirm it.

## Consent

Written informed consent for the publication of the case report and any associated images was obtained from the patient.

## Data availability

All data underlying the results are available as part of the article and no additional source data are required.
