# Supplementary material for: Case Report: Dilated cardiomyopathy with biventricular thrombus secondary to impaired coagulation in a patient with HIV
Source: F1000Res. 2020 Jul 7;9:610. Originally published 2020 Jun 15. [Version 2] doi: 10.12688/f1000research.24016.2 (PMC7361504; doi:10.12688/f1000research.24016.2)
Supplement: Peer review history

# Peer review: Reviewer response for version 2

**DOI:** 10.5256/f1000research.27810.r66622

**Published:** 2020-07-14

**Authors:** Satyanarayana Vaidya

**Affiliations:** 1 Emory University, Atlanta, GA, USA

The authors have addressed most of major concerns in the case report. The case report emphasizes the importance of considering increased thrombogenecity in HIV patients.

Are enough details provided of any physical examination and diagnostic tests, treatment given and outcomes?

Partly

Is the case presented with sufficient detail to be useful for other practitioners?

Partly

Is sufficient discussion included of the importance of the findings and their relevance to future understanding of disease processes, diagnosis or treatment?

Yes

Is the background of the case’s history and progression described in sufficient detail?

Yes

Reviewer Expertise:

Nephrology

I confirm that I have read this submission and believe that I have an appropriate level of expertise to confirm that it is of an acceptable scientific standard.

# Peer review: Reviewer response for version 2

**DOI:** 10.5256/f1000research.27810.r66621

**Published:** 2020-07-08

**Authors:** Raju Vaddepally

**Affiliations:** 1 Department of Hematology/Medical Oncology, Yuma Regional Medical Center, Yuma, AZ, USA

Are enough details provided of any physical examination and diagnostic tests, treatment given and outcomes?

Yes

Is the case presented with sufficient detail to be useful for other practitioners?

Yes

Is sufficient discussion included of the importance of the findings and their relevance to future understanding of disease processes, diagnosis or treatment?

Partly

Is the background of the case’s history and progression described in sufficient detail?

Yes

Reviewer Expertise:

Hematology, Medical Oncology Thoracic and head and neck medical Oncology, Immunotherapy, Targeted therapy, Precision Medicine.

I confirm that I have read this submission and believe that I have an appropriate level of expertise to confirm that it is of an acceptable scientific standard.

# Peer review: Reviewer response for version 1

**DOI:** 10.5256/f1000research.26492.r64869

**Published:** 2020-07-01

**Authors:** Satyanarayana Vaidya

**Affiliations:** 1 Emory University, Atlanta, GA, USA

Authors have done a commendable job in writing a detailed case report depicting Increased thrombogenicity and a low blood flow state in HIV a infected patient as primary mechanisms of the virchow triad causing biventricular thrombosis. The tables, figures provide great deal of detailed information regarding the location of thrombus.

 I would like to highlight certain important points and would encourage the inclusion of those in the case report

1. The low flow state due to cardiomyopathy seems to have been a primary mechanism here causing biventricular thrombosis. If patient had a transesophageal echocardiography to exclude a patent foramen ovale which can result in paradoxical embolism or deep vein assessment with a venous doppler, inclusion of that information would be critical to exclude any possibility of venous thromboembolism
2. Patient seems to have a decent CD4 count of 815 cells/microliter despite being non-compliant with Antiretroviral therapy. Inclusion of HIV viral load would give more information as there is evidence regarding high viral loads and low CD4 counts being related to increased thrombogenicity. In addition, presence of a reasonable CD4 cell count argues against the possibility of HIV associated nephropathy which can cause significant proteinuria causing loss of anticoagulant proteins causing a thrombogenic state. Information regarding protein: creatinine ratio would have evaluated that possibility.
3. One would always make an argument of low protein C, protein S levels being low in the setting of acute thrombosis. Following them after a period of at least 6 months would reveal the true levels and differentiate between true deficiency vs due to consumption due to thrombosis.

Are enough details provided of any physical examination and diagnostic tests, treatment given and outcomes?

Partly

Is the case presented with sufficient detail to be useful for other practitioners?

Partly

Is sufficient discussion included of the importance of the findings and their relevance to future understanding of disease processes, diagnosis or treatment?

Yes

Is the background of the case’s history and progression described in sufficient detail?

Yes

Reviewer Expertise:

Nephrology

I confirm that I have read this submission and believe that I have an appropriate level of expertise to confirm that it is of an acceptable scientific standard, however I have significant reservations, as outlined above.

# Response

**Published:** 2020-07-02

**Authors:** Chetan Kammari

**Affiliations:** Cape fear valley Hospital, USA

We agree with the reviewer's comment that the low flow state due to cardiomyopathy could have contributed, we think it is multifactorial in this patient with low flow state as well as possible protein deficiencies if truly positive contributing to it. Unfortunately, a TEE was not done during that hospitalization.

 I agree with the reviewer's comment that low protein C, low protein S, and anti-thrombin deficiency could represent the state of thrombus consuming the factors. It is not 6 months yet and the patient continues to be followed up as an outpatient.  Modified in the manuscript.

 We thank the reviewer for pointing out normal CD4 count - low CD4 count is one of the risk factors, but there additional risk factors in HIV infected patients like a protein C deficiency, protein S deficiency, antithrombin deficiency, antiphospholipid antibody syndrome, endothelial dysfunction in addition to the traditional risk factors such as obesity, cigarette smoking, hypertension, immobilization.  We think that thrombus is multifactorial in this patient.  We did not think the patient has HIV-induced nephropathy resulting in nephrotic syndrome - hence it was not investigated further, no protein creatinine ratio was done during the admission.

# Peer review: Reviewer response for version 1

**DOI:** 10.5256/f1000research.26492.r64868

**Published:** 2020-06-29

**Authors:** Raju Vaddepally

**Affiliations:** 1 Department of Hematology/Medical Oncology, Yuma Regional Medical Center, Yuma, AZ, USA

This is a very nice case review on alterations in homeostatic mechanism in HIV. I tend to agree that there are inflammatory changes in HIV patients that deranges the the coagulation equilbrium and there are limitations in available literature on what exactly drives the prothrombotic features in such patients. 

 Having said that in this case, the patient seems to have dilated cardiomyopathy which largely seems to have driven the thrombosis and the case has been very well elaborated with description, pictures, and lab assay. However, the low protein C, S, and Antithrombin evidenced here rather depicts the state of thrombus consuming the factors. It would have been nice if they had follow up lab assay follow on these protein C, S, and Antithrombin and if the numbers continue to remain low after 3-6 months from the time of thrombosis then one could make a strong argument that the coagulation factor deficiencies could have accentuanted the thrombosis but it is unclear at this time based on the available labs.

 The authors should be commended for ensuring the APAS etiology has been ruled out which would be an important etiology in HIV patients. However, I do suggest trimming the case report content especially in the first paragraph where there is redundancy in non-pertinent information. Overall, I feel this contributes some knowledge to existing data of HIV and hypercoagulability.

Are enough details provided of any physical examination and diagnostic tests, treatment given and outcomes?

Yes

Is the case presented with sufficient detail to be useful for other practitioners?

Yes

Is sufficient discussion included of the importance of the findings and their relevance to future understanding of disease processes, diagnosis or treatment?

Partly

Is the background of the case’s history and progression described in sufficient detail?

Yes

Reviewer Expertise:

Hematology, Medical Oncology Thoracic and head and neck medical Oncology, Immunotherapy, Targeted therapy, Precision Medicine.

I confirm that I have read this submission and believe that I have an appropriate level of expertise to confirm that it is of an acceptable scientific standard, however I have significant reservations, as outlined above.

# Response

**Published:** 2020-07-02

**Authors:** Chetan Kammari

**Affiliations:** Cape fear valley Hospital, USA

I agree with the reviewer's comment that low protein C, low protein S, and anti-thrombin deficiency could represent the state of thrombus consuming the factors. It is not 6 months yet and the patient continues to be followed up as an outpatient. Modified in the manuscript.

 Modified in the manuscript as below - We think that low protein C, low protein S levels, and antithrombin-III deficiency could contribute to thrombus formation if truly positive in addition to the risk factors discussed above. However, it could represent a thrombus consuming the factors, and they will be repeated six months to confirm it.

 We trimmed the case report content in the first paragraph as advised.

 First Paragraph -

 The patient is a 57-year-old Caucasian male with a known past medical history of the human immunodeficiency virus (HIV) non-compliant with medical therapy and hyperlipidemia, who presented to the emergency department with shortness of breath, hypoxia with oxygen saturation of 70%, pleuritic chest pain and a syncopal episode with fall.  The patient denied any significant family, surgical, or social history. He was treated for pneumonia six weeks before presentation with antibiotics, and since then, he has been experiencing exertional dyspnea. Patient unable to do his activities of daily living due to exertional dyspnea. The patient denied orthopnea or paroxysmal nocturnal dyspnea. He had a syncopal episode at home with fall resulting in left pleuritic chest pain. The patient admitted that he had previous syncope episodes that occur with little or no warning signs except for mild dizziness before passing out.  The physical examination was significant for chest wall tenderness with a normal cardiorespiratory exam.
